# A Method for Choosing the Best Samples for Mars Sample Return

**DOI:** 10.1089/ast.2017.1744

**Published:** 2018-05-01

**Authors:** Peter R. Gordon, Mark A. Sephton

**Affiliations:** Impacts and Astromaterials Research Centre, Department of Earth Science and Engineering, Imperial College London, UK.

## Abstract

Success of a future Mars Sample Return mission will depend on the correct choice of samples. Pyrolysis-FTIR can be employed as a triage instrument for Mars Sample Return. The technique can thermally dissociate minerals and organic matter for detection. Identification of certain mineral types can determine the habitability of the depositional environment, past or present, while detection of organic matter may suggest past or present habitation. In Mars' history, the Theiikian era represents an attractive target for life search missions and the acquisition of samples. The acidic and increasingly dry Theiikian may have been habitable and followed a lengthy neutral and wet period in Mars' history during which life could have originated and proliferated to achieve relatively abundant levels of biomass with a wide distribution. Moreover, the sulfate minerals produced in the Theiikian are also known to be good preservers of organic matter. We have used pyrolysis-FTIR and samples from a Mars analog ferrous acid stream with a thriving ecosystem to test the triage concept. Pyrolysis-FTIR identified those samples with the greatest probability of habitability and habitation. A three-tier scoring system was developed based on the detection of (i) organic signals, (ii) carbon dioxide and water, and (iii) sulfur dioxide. The presence of each component was given a score of A, B, or C depending on whether the substance had been detected, tentatively detected, or not detected, respectively. Single-step (for greatest possible sensitivity) or multistep (for more diagnostic data) pyrolysis-FTIR methods informed the assignments. The system allowed the highest-priority samples to be categorized as AAA (or A*AA if the organic signal was complex), while the lowest-priority samples could be categorized as CCC. Our methods provide a mechanism with which to rank samples and identify those that should take the highest priority for return to Earth during a Mars Sample Return mission. Key Words: Mars—Astrobiology—Search for Mars' organics—Infrared spectroscopy—Planetary habitability and biosignatures. Astrobiology 18, 556–570.

## 1. Introduction

Attempts to discover whether life exists or has previously existed on Mars have not yet been conclusive. To seek greater certainty when reading the rock record of habitability and habitation on Mars, mission concepts are being proposed that involve the return of samples to Earth (McLennan *et al.,* 2012). Once on Earth, samples of Mars can be distributed to multiple laboratories that host the most powerful analytical techniques available. The probability of successfully obtaining conclusive evidence of past or present life on Mars is inevitably influenced by the choice of sample to be returned to Earth (Sephton and Carter, 2015). Consequently, attention has been directed toward methods that triage the range of possible sampling opportunities on Mars to effectively select a few highest-priority samples.

Environments on Mars that are believed to have been wet (Carr, 1996) are the primary locations for life searches (McLennan *et al.,* 2012). Sedimentary features imply the presence of liquid water to produce alluvial fans (Malin and Edgett, 2000), distributary fans (Malin and Edgett, 2003; Bhattacharya *et al.,* 2005; Lewis *et al.,* 2008), and paleolakes (Irwin *et al.,* 2005). Hydrated minerals represent evidence of water on early Mars (Bibring *et al.,* 2006; Poulet *et al.,* 2005). Isotopic evidence suggests the presence of a thicker atmosphere in the past (Owen and Bar-Nun, 1995). The duration of equable conditions probably extended into the era when volcanic emissions and the loss of atmosphere led to increasingly acidic and saline chemistries (Bibring *et al.,* 2006). There have been some suggestions that wet environments may still be found on present-day Mars. Recurring slope lineae are an observed phenomenon on Mars, and while now thought to be better explained by dry sand flows (Dundas *et al.,* 2017), they have been interpreted as the result of present-day water-containing flows (Ojha *et al.,* 2015), while other proposals to explain these features include subsurface water reservoirs that produced surface outflows from late Hesperian to the present day (Fassett *et al.,* 2010; Rodríguez *et al.,* 2010; Bramson *et al.,* 2015).

The geological history of Mars is generally described by three main eras, namely, the Noachian, Hesperian, and Amazonian, for which ages are informed by crater density studies. Mineralogical mapping data from the Mars Express orbiter has led to a proposed complementary timescale that reflects the dominant mineralogies present on Mars (Bibring *et al.,* 2006). The new timescale provides immediate mineralogical context in reference to one of the following eras: the Phyllocian, Theiikian, or Siderikian, which reflect abundant phyllosilicates, sulfates, and iron oxides, respectively. If life emerged on Mars, then this event most likely occurred in the Phyllocian era when wet and neutral conditions were prevalent and conditions were most similar to those on present-day Earth. It is reasonable to consider that any Phyllocian life would have persisted for the remainder of the era and experienced the transition to the Theiikian era when wet and neutral settings were replaced with wet and acidic environments (Bibring *et al.,* 2006). It is also logical to assume that by the beginning of the Theiikian any life that originated in the Phyllocian would have evolved and proliferated to achieve the greatest possible abundance of biomass and its widest distribution. The Early Theiikian biomass would have been available for preservation in the martian rock record. The acidic conditions in the Theiikian led to the widespread deposition of sulfate minerals, and sulfate-rich environments are known to sustain life and offer high potential for organic matter preservation (Farmer and Des Marais, 1999).

Developing triage methods for use on Mars is hindered by the lack of readily accessible martian samples. No samples have been returned from Mars by space missions, and meteorites from Mars are precious and present in relatively small amounts. Testing of Mars triage methods must therefore rely on the use of analog sites and samples. Mineralogical samples such as the glassy volcanic ash JSC Mars-1 allow the testing of equipment destined for use on the basalt-rich martian surface (Allen *et al.,* 1997). Astrobiology studies seek out Mars-like conditions that include dry areas such as the Atacama Desert or cold environments such as the Antarctic Dry Valleys (Marlow *et al.,* 2011; Preston and Dartnell 2014). Yet the analogues most relevant to the early Theiikian are highly acidic rivers and streams often reflecting the aqueous oxidation of pyrite, a celebrated example of which is Río Tinto, Spain (Fernández-Remolar *et al.,* 2005).

One instrument that has been proposed for triage on Mars is pyrolysis-FTIR (Sephton *et al.,* 2013). Fourier transform infrared (FTIR) is relatively simple in operation, has a limited demand for resources, produces richly diagnostic information, and has been considered for use on Mars previously (Anderson *et al.,* 2005). Pyrolysis-FTIR negates the mechanical sample loading challenges associated with other solid phase FTIR techniques by adding a thermal extraction technique that has been successfully deployed on numerous missions on which pyrolysis ovens were used, namely Viking 1 and 2, Phoenix, and Mars Science Laboratory (Biemann *et al.,* 1976; Hoffman *et al.,* 2008; Mahaffy *et al.,* 2012). During pyrolysis-FTIR, samples are rapidly heated (up to 20,000°C s^−1^) to produce volatiles that are subsequently detected and characterized by infrared spectroscopy. An effective triage method should prioritize samples that display evidence of a life-supporting environment, contain organic compounds, and if possible suggest that those organic compounds are complex and therefore information-rich in nature. Pyrolysis-FTIR has been demonstrated to provide diagnostic information on the mineralogy of samples and therefore past habitability (Gordon and Sephton, 2016b). Pyrolysis-FTIR has also been utilized to provide insights into the probability of the presence of biosignatures, demonstrating that detection of organic compounds in concentrations as low as tens of parts per million is achievable (Gordon and Sephton 2016a).

In this paper, we apply pyrolysis-FTIR to a Mars analog sample set. Ferrous sulfate–rich streams are found on the southern coast of England where oxidation of sedimentary pyrite produces acidic waters. The sulfate-rich streams support a vibrant ecosystem of acid-tolerant species and provide effective analogues for the Theiikian of Mars. We use the sulfate ecosystem samples and pyrolysis-FTIR to emulate the sample triage and selection process as could be operated on Mars. In the case of an actual mission, the triage process would be preceded by complementary steps, such as imaging or spectroscopy; however, this study aims to demonstrate the triage capability of pyrolysis-FTIR when used in isolation. Our findings suggest an effective method for choosing those samples which would provide the highest possibility of success when returned to Earth for life-detection analyses.

## 2. Methods

### 2.1. Sample selection

**FIG. 1.**
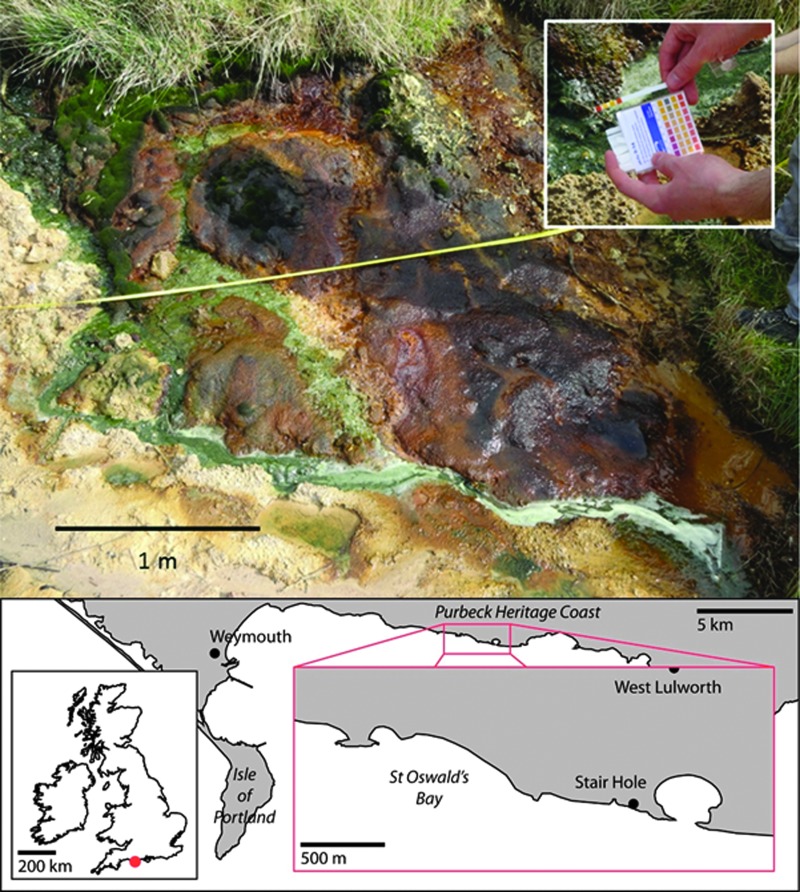
A ferrous sulfate–rich stream in Dorset, southern England. Oxidation of pyrite gives the water a pH of 3.5 (inset). Jarosite is precipitated, and where water volumes and/or pH increase, the jarosite is converted to goethite. A map is included (bottom) showing the locations of the sampling regions, St Oswald's Bay and Stair Hole, within the United Kingdom.

Samples were obtained from two acidic, ferrous sulfate–rich streams located in Dorset, southern England: one characterized by flowing water (Fig. 1) and one that was dry (Lewis *et al.,* 2018). The collected sample set is listed in Table 1. The majority of samples collected came from the flowing stream, which was located at St Oswald's Bay, while the dry stream was to the east of St Oswald's Bay, in a small cove known as Stair Hole. Oxidation of pyrite, abundant in the Wealden Beds from which the St Oswald's Bay stream flows, gives the water a pH of 3.5, and jarosite deposits accumulate where the ferrous sulfate–rich waters evaporate. The dry stream at Stair Hole was less acidic, with a pH level of 5. Lateral variations across the steam section were significant. Where water volumes and/or pH increased, jarosite was converted to goethite; and where the stream was relatively dry, a jarosite-containing quartz sand occurred. The goethite was covered by a purple microbial mat, and the deepest parts of the stream contained acidophilic algae. The sample codes listed in Table 1 are descriptive with, for example, a flowing stream sample with wood over goethite-rich minerals being called “FlowWG” and a dry stream containing a microbial mat over jarosite-rich minerals being called “DryMJ.” Stream samples were obtained as cores before being freeze-dried and crushed in preparation for analysis. Details of sample collection and preparation are present in a previously published paper (Lewis *et al.,* 2018).

**Table 1.** Samples from Flowing and Dry Acidic, Ferrous Sulfate–Rich Streams

*Sample*	*Code*	*Hand specimen description*	*Distance from west bank (cm)*	*Distance from center (cm)*	*pH*	*Max potential gas products by stoichiometry (%)*
*Flowing stream*						
Bank sediment (West)	FlowBS1	Quartz sand with some clay minerals and jarosite	0	−225	5	w:2.3, s:0.1
	FlowBS2	Quartz sand with clay minerals and some jarosite	30	−195	4.5	w:3.9, s:2.2
Matt over goethite	FlowMG1a	Microbial mat over quartz sand with goethite with some jarosite	85	−140	4	w:3.7, s:2.6
	FlowMG1b	Microbial mat over quartz sand with abundant goethite and some jarosite				w:7.8, s:1.2
	FlowMG1c	Microbial mat over quartz sand with some jarosite and clay minerals				w:1.1, s:1.2
	FlowMG2a	Microbial mat over quartz sand with abundant goethite and some clay minerals	150	−75	5.5	w:6.2, s:0.0
	FlowMG2b	Microbial mat over quartz sand with abundant goethite				w:4.8, s:0.0
	FlowMG2c	Microbial mat over quartz sand with some jarosite and clay minerals				w:1.2, s:1.6
Wood over goethite	FlowWG1a	Wood in quartz sand with abundant goethite	190	−35	4.5	w:4.6, s:0.0
	FlowWG1b	Microbial mat over quartz sand with some jarosite and clay minerals				w:1.4, s:1.8
Matt over jarosite	FlowMJ1a	Microbial mat over quartz sand with abundant goethite and some jarosite and clay minerals	225	0	4	w:3.6, s:0.4
	FlowMJ1b	Microbial mat over quartz sand				w:0.0, s:0.0
	FlowMJ1c	Microbial mat over quartz sand with abundant jarosite				w:2.8, s:6.7
Wood over jarosite	FlowWJ1a	Wood in quartz sand with abundant jarosite and some clay minerals Q:64.9, G:0, J:27, I:0, K:8.1, M:0	260	35	5	w:4.2, s:6.9
	FlowWJ1b	Wood in quartz sand with abundant jarosite and some clay minerals Q:63.2, G:0, J:27.5, I:0, K:9.3, M:0				w:4.4, s:7.0
Bank sediment (East)	FlowBS3	Quartz sand with abundant clay minerals and some jarosite	325	100	4	w:2.5, s:0.3
Quartz sand	FlowQ1	Quartz sand with some clay minerals Q:87.4, G:0, J:0.5, I:6.9, K:3.4, M:1.8	380	155	4	w:0.9, s:0.1
*Dry stream*						
	DryMJ1a	Microbial mat over quartz sand with abundant goethite and some jarosite and clay minerals Q:40.3, G:18, J:5.5, I:25.3, K:10.9, M:0			5	w:5.3, s:1.4
	DryMJ1b	Microbial mat over quartz sand with abundant goethite and jarosite				w:5.9, s:4.9

Sample codes are prefixed by “Flow” or “Dry” to indicate whether they were extracted from the flowing and dry stream, respectively. The following two letters describe the general nature of the sample: either BS (bank sediment), or some combination of M (microbial mat) or W (wood) over G (goethite) or J (jarosite). The number suffixes distinguish additional cores taken for similar sample types, while the lettered suffix indicates the stratigraphic/vertical position of a sample within the same core (with “a” being the top-most sample). Stoichiometry abbreviations are as follows: w = water; s = sulfur dioxide.

### 2.2. Attenuated total reflectance–FTIR

All sulfate stream samples were analyzed by using a Thermo Nicolet 5700 FTIR spectrometer fitted with an attenuated total reflectance (ATR) Thermo Orbit accessory to provide supporting characterization data for the samples. These data revealed the chemical nature of the samples but were not used in the triage process, which utilized pyrolysis-FTIR exclusively. Note that these data did not influence the selection of samples in the triage process, and similar ATR data need not be acquired on Mars. Powdered samples were pressed against the diamond ATR crystal and spectra collected by using a method aggregating 128 sample scans over a 150 s period at a resolution of 4 cm^−1^, from which a background scan (*i.e.,* a spectrum taken of the crystal platform with no sample present) was subtracted. Resulting spectra were then processed with an automatic baseline correction method provided by the Thermo Scientific OMNIC Software Suite, which attempts to account for the effects of optical depths varying as a function of wavelength. Spectral features arising from hydroxyl, water of hydration, carbonates, sulfates, and organic compounds were identified in the ATR-FTIR spectra by using the same criteria as described in a previous investigation (Gordon and Sephton, 2016b).

### 2.3. Pyrolysis-FTIR

Each pyrolysis sample was prepared by adding a quantity (in the range 4–23 mg) of the chosen sample type to a quartz tube with the powder being held in place by quartz wool plugs at both ends. Mass measurements were taken, on a scale accurate to 0.1 mg, during the sample preparation steps so that the mass of powdered sample could be determined.

Pyrolysis was achieved with a CDS Analytical Pyroprobe 5200. The pyroprobe is a length of metal rod with a platinum coil heating element at one end. The quartz tube containing the sample was loaded into the platinum coil before the probe was inserted into a gas-tight CDS Analytical Brill Cell, which provided an interface with the FTIR spectrometer (ZnSe windows at two ends of the cell permit an infrared beam to traverse the intermediate cell volume). Pyrolysis was achieved when the coil was heated at a controlled rate of 20,000°C s^−1^ and then held at the desired temperature for 7.2 s. Gas products liberated from the solid sample were contained within the helium atmosphere of the Brill Cell. A controlled helium flow was used to purge the cell of spent pyrolysis products between analyses.

Fourier transform infrared analysis was conducted with a Thermo Scientific Nicolet 5700 FTIR spectrometer. Before pyrolysis, a background scan was taken with the probe loaded in the purged cell. The sample spectrum was taken immediately after the probe had finished its rapid heating step. Background and sample spectra were composed of 32 scans taken over 19.5 s at a resolution of 4 cm^−1^. To account for background artifacts introduced by the experimental process, blanks were prepared in the same manner as geological samples. Before each collection session, three procedural blanks were analyzed at each of the temperature modes used for analysis. For each sample spectrum obtained, an average of the appropriate procedural blank spectra was subtracted. The standard deviation of procedural blanks was used to calculate the 95% confidence interval (*i.e.,* 2σ), used to represent the uncertainty in measurements.

Measurements of spectral features were taken and recorded: a peak located at 2349 cm^−1^ corresponding to the anti-symmetric stretch in carbon dioxide, one at 3853 cm^−1^ arising from a stretching mode of water, one at 1352 cm^−1^ corresponding to the sulfur dioxide anti-symmetric stretching mode, one at 3016 cm^−1^ corresponding to the methane anti-symmetric stretching mode and the height of a peak at 2933 cm^−1^. Organic compounds, depending on the molecular composition and structure, typically produce features in a broad spectral window corresponding to the C-H stretch; for comparison with a previous study (Gordon and Sephton, 2016a), the region (3150–2740 cm^−1^) was chosen to represent organic compounds. The mass of methane, water, carbon dioxide, and sulfur dioxide products were determined by reference to mass calibration curves. Spectrometer operation and data processing were both achieved with the Thermo Scientific OMNIC Software Suite.

### 2.4. Triage operation

To identify samples of highest priority for biosignature detection, a triage methodology was developed. A phased approach was adopted to utilize the advantages of different modes of pyrolysis-FTIR (the advantages of the chosen modes had been determined in previous investigations [Gordon and Sephton 2016a, 2016b]). The phases were designed to occur in a sequence: an initial habitability assessment phase, a subsequent habitation assessment phase, and a final diagnostic phase. A scoring system was introduced to allow discernment and ranking of samples. Revision of a sample score can occur as new information from subsequent phases becomes available. All samples were subjected to the first two phases, and some low-priority samples were analyzed in the third phase just to verify the triage method; in actual field operation, samples would only be promoted to subsequent phases based on their favorable responses to pyrolysis-FTIR. Individual triage phases may indicate different scores for aliquots of the same sample. In these cases, the sample maintains the highest rank achieved even if the higher rank was awarded in the previous phase.

#### 2.4.1. Triage phase one (the habitability assessment phase)

All samples were first subjected to a single-step 1000°C pyrolysis-FTIR analysis. This mode was identified in previous work as a high-sensitivity method for detecting geological indicators of habitability (Gordon and Sephton, 2016b). Certain minerals indicative of past habitability, such as serpentinites and carbonates, only release their identifying gases at high temperatures. At high temperatures, organic compounds are more prone to complete thermal dissociation, and their products consolidate into a methane signal. Organic compounds also undergo more aggressive combustion at higher temperatures owing to the greater energy input and interaction with any oxidants which arise from decomposition of the mineral matrix than at lower temperatures. However, combusted organic compounds are revealed through the simultaneous release of combustion products (carbon dioxide and water). Thus, the 1000°C single step acts as the triage “catch all” phase, providing good general-purpose sensitivity but lacking more detailed diagnostic information.

#### 2.4.2. Triage phase two (the habitation assessment phase)

The highest-ranking sample types of the habitability assessment (single-step 1000°C) phase were passed to a subsequent round of single-step 700°C pyrolysis-FTIR analyses. A temperature of 700°C avoids the extensive organic compound thermal dissociation of the higher 1000°C step and is more sensitive to detection and identification of complex organic compounds. Although during an actual triage operation only the highest-ranking samples would go forward to the habitation (single-step 700°C) assessment, in this study all samples were analyzed at both 700°C and 1000°C to test the efficacy of the triage methodology.

#### 2.4.3. Triage phase three (the diagnostic phase)

The highest-ranking sample types of the previous phases were then passed to multistep pyrolysis-FTIR analysis (successive analysis steps performed at 500°C, 750°C, and 1000°C). This form of pyrolysis-FTIR provides additional diagnostic information relative to single-step analysis. The characteristic decomposition temperatures and the ratio of gases released at the different temperature steps are characteristic for each mineral phase (Gordon and Sephton, 2016b). Comparing these with preexisting reference spectra can help identify the material type *in situ*. Although, as explained above, during an actual triage operation only the highest-ranking samples from single-step analysis would be used further for multistep analysis, in this study a number of low-priority samples were also processed in the multistep phase to test the accuracy of the triage methodology.

### 2.5. Classifying and ranking sample potential

**FIG. 2.**
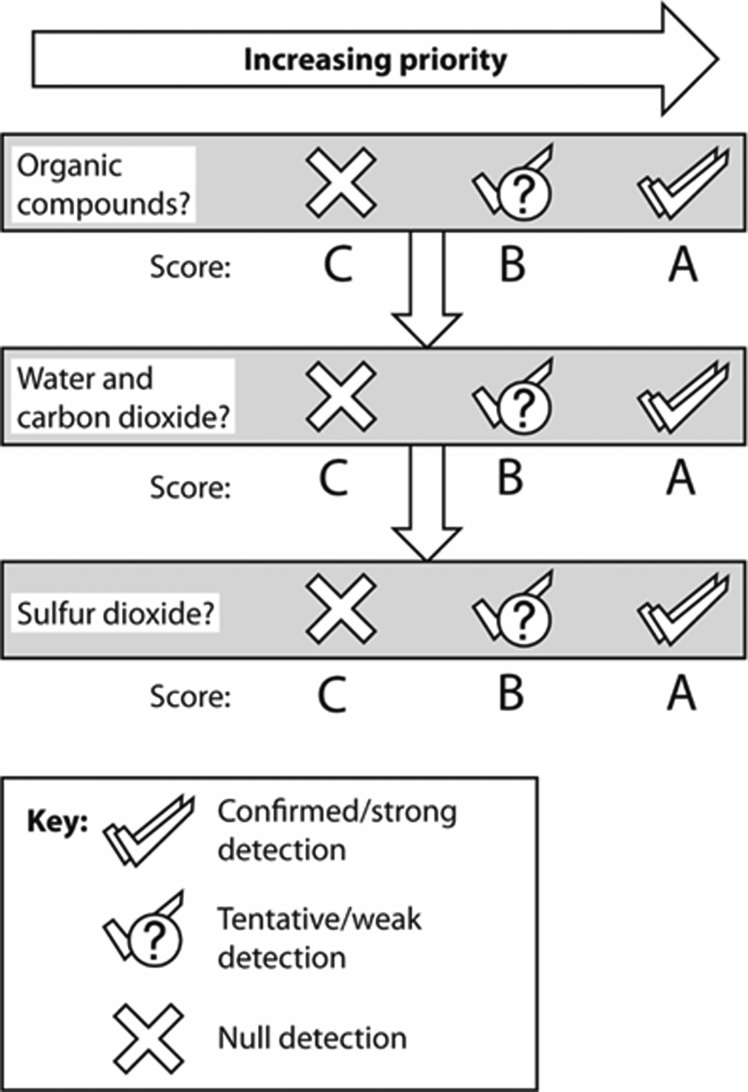
Logic for scoring samples for the purpose of ranking them.

In the aim of maximizing resource efficiency, it is not intended, in the case of an actual mission, that samples of low scientific value will be processed by the full set of triage phases described above. To discover the samples that should be processed further following single-step analyses, a scoring logic was applied to the pyrolysis-FTIR outcomes. This process is illustrated in Fig. 2 and described in detail below.

Three tiers of assessment were used to construct a triage code for each sample. Each part of the code is determined by the response of the FTIR signals representing that tier (described below in Sections 2.5.1, 2.5.2, and 2.5.3). Before a sample is scored using the criteria of each tier, the presence of a signal is considered, the thresholds for which were set to discern strongly against false positives and are as follows:

C. Confirmed—signal over double the associated uncertainty.
T. Tentative—signal greater than the associated uncertainty but weaker than double.
N. None—signal less than uncertainty.

Because the objective of Mars Sample Return is the detection of evidence of life, the importance of the tiers decreases from Tier 1 to Tier 3. Tier 1 represents direct organic responses, Tier 2 represents the gaseous products of organic degradation or indicators or habitability, while Tier 3 represents gaseous indicators of habitability. Tier 3 also provides information on the presence of sulfates, which are a recognized organic signal attenuator (owing to the generation of oxidants during heating and the corresponding combustion of organic compounds present), and their presence suggests that any organic detection in Tier 1 can only be a minimum value.

#### 2.5.1. Tier 1—Organic compound response

This is the highest-priority tier. Owing to the great importance of organic matter as evidence of life, complex organic compounds are preferred over methane. Where complex organic responses are present instead or in addition to methane, therefore, they are indicated by modifying the A with a superscript (*).

A. Confirmed organic compounds (A*) or methane signal (A).
B. Tentative organic compounds or methane signal.
C. No organic compounds or methane detected.

#### 2.5.2. Tier 2—Simultaneous water and carbon dioxide response

Individually, these gases serve as habitability indicators; however, a simultaneous release (*i.e.,* both gases present in the spectrum resulting from a pyrolysis-FTIR analysis) introduces the possibility of combusted organic compounds. With carbon dioxide and water having other possible non-organic sources, an additional criterion is applied: both carbon dioxide and water contents must exceed 2% of the initial sample weight to be considered a strong signal. In a previous investigation (Gordon and Sephton, 2016a), mineral types representative of the Phyllocian and Theiikian, the martian eras considered most habitable, only produced simultaneous releases of water and carbon dioxide above 2% of initial sample mass when organic compounds were present in the sample.

A. Confirmed and strong (above 2% of sample mass) water and carbon dioxide signals.
B. Tentative or confirmed but low (equal or less than 2% of sample mass) water and carbon dioxide signals.
C. No simultaneous release of water and carbon dioxide.

#### 2.5.3. Tier 3—Sulfur dioxide response

As sulfates have been recognized for aiding the preservation of biosignatures (Aubrey *et al.,* 2006), samples presenting sulfur dioxide are desirable. In addition, sulfur dioxide has been seen to assist the combustion of organic compounds when heated (Lewis *et al.,* 2014); thus when a sulfur signal is present, it is likely that any accompanying hydrocarbon signal represents a minimum response for the organic richness of the original sample and provides encouragement for any organic compound detection in Tier 1 or corroboration for any organic combustion signature in Tier 2.

A. Confirmed sulfur dioxide signal.
B. Tentative sulfur dioxide signal.
C. No sulfur dioxide.

In summary, a sample scoring A*AA would be the highest-priority sample and CCC the lowest. Two samples that are assigned the same code can be ranked against each other by (i) prioritizing the sample with a response for complex organic compounds and (ii) providing a higher rank to the samples with greatest responses in the highest-priority tier (*i.e.,* Tier 1 > Tier 2 > Tier 3).

## 3. Results

### 3.1. Attenuated total reflectance-FTIR

The results from ATR-FTIR analysis are presented in Table 2. A broad peak for water of hydration was observed for all samples and detected in weak form for two samples from the flowing stream containing microbial mat over jarosite and quartz sand (FlowMJ1b and FlowQ1), while it was detected strongly in all other samples. Hydroxyl peaks were observed in strong form for wood-containing samples and bank sediments from the flowing stream and a microbial mat–containing sample from the dry stream (FlowWJ1b, FlowBS1, FlowBS2, FlowBS3, DryMJ1a), and they were observed in weak form for wood-, microbial mat–, and quartz sand–containing samples from the flowing stream (FlowWG1a, FlowWG1b, FlowMG1c, FlowMJ1a, FlowMJ1b, FlowQ1). Some samples exhibited a broad peak around 1400 cm^−1^, which is characteristic of carbonates, yet they cannot be conclusively assigned as such because the other characteristic carbonate peaks at 890–800 cm^−1^ and 760–670 cm^−1^ were not conclusively identifiable. Thus, samples exhibiting a broad 1400 cm^−1^ peak were identified to have organic matter because alkanes, alcohols, and amides can have strong responses in this region. A feature representing sulfates could be identified in all samples in the region of 1090 cm^−1^. In wood-, microbial mat–, and quartz sand–containing samples and bank sediments from the flowing stream (FlowWG1b, FlowMG2a, FlowMG2b, FlowMJ1a, FlowMJ1b, FlowQ1, FlowBS1), the 1090 cm^−1^ response only came in the form of a small shoulder, which was insufficient to conclusively determine the presence of sulfates. Sulfates could be determined weakly in wood-containing samples and bank sediments from the flowing stream (FlowWG1a, FlowBS3) but strongly in all other samples. Organic compounds were indicated by broad features arising from C-H stretching in the 3050–2650 cm^−1^ region, and they were observed strongly in wood- and microbial mat–containing samples from the flowing and dry streams (FlowWG1a, FlowMG1a, FlowMG2a, FlowMJ1a, DryMJ1a) and weakly in wood- and microbial mat–containing samples from the flowing and dry streams and bank sediments from the flowing stream (FlowWG1b, FlowWJ1b, FlowMG1b, FlowMG2b, DryMJ1b, FlowBS1, FlowBS2). A small sharp peak at 3020 cm^−1^ representing C-H stretching was observed in spectra from wood and microbial mat samples from the flowing and dry streams (FlowWG1a, FlowWJ1b, FlowMG1b, FlowMG2b, DryMJ1a, and DryMJ1b).

**Table 2.** ATR-FTIR Results

*Sample*	*Code*	*Hydroxyl*	*Water of hydration / adsorbed water*	*Carbonate ion*	*Sulfate ion*	*Organic compounds*
*Flowing stream*						
Bank sediment (W)	FlowBS1	■	■		?	□
	FlowBS2	■	■		■	□
Matt over goethite	FlowMG1a		■	?	■	■
	FlowMG1b		■		■	□
	FlowMG1c	□	■		■	
	FlowMG2a		■	?	?	■
	FlowMG2b		■		?	□
	FlowMG2c		■		■	
Wood over goethite	FlowWG1a	□	■	?	□	■
	FlowWG1b	□	■		?	□
Matt over jarosite	FlowMJ1a	□	■	?	?	■
	FlowMJ1b	□	□		?	
	FlowMJ1c		■		■	
Wood over jarosite	FlowWJ1a		■		■	
	FlowWJ1b	■	■		■	□
Bank sediment (E)	FlowBS3	■	■		□	
Quartz sand	FlowQ1	□	□		?	
*Dry stream*						
	DryMJ1a	■	■	?	■	■
	DryMJ1b		■		■	□

Solid squares represent strong identification, while an empty square represents a relatively weak signal. A question mark is used to denote samples that exhibit a spectral feature in the characteristic region yet cannot be conclusively assigned: a broad peak around 1400 cm^−1^ in the case of carbonates and a shoulder at around 1090 cm^−1^ in the case of sulfates.

### 3.2. Triage phase one (the habitability assessment phase) using single-step pyrolysis-FTIR (1000°C)

**FIG. 3.**
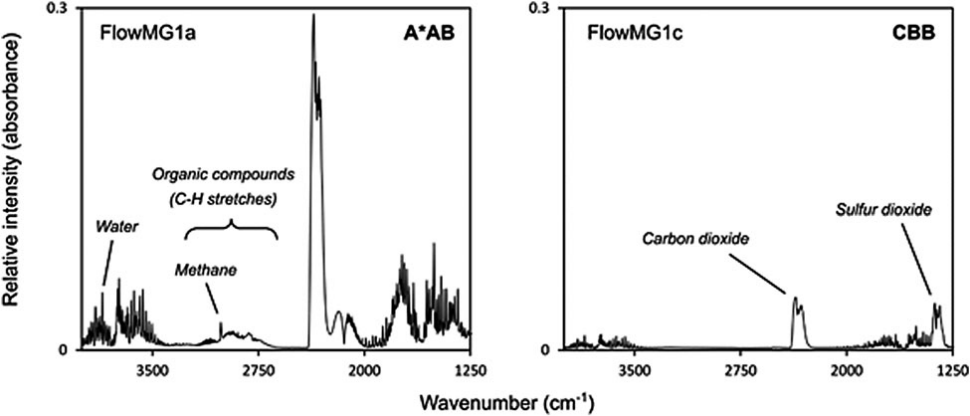
Example pyrolysis-FTIR spectra, showing gas responses for the highest- and lowest-ranking samples, FlowMG1a and FlowMG1c respectively, in the first triage phase (1000°C single-step pyrolysis-FTIR).

Results from the first triage phase (1000°C single-step pyrolysis-FTIR) are presented in Table 3. Figure 3 shows an example pyrolysis-FTIR spectra, containing gas responses for the highest- and lowest-ranking samples, FlowMG1a and FlowMG1c, respectively. Hydrocarbon responses were confirmed in the wood- and microbial mat–containing samples from flowing and dry streams (FlowWG1a, FlowMG1a, FlowMJ1a, FlowMG2a, FlowMG2b, DryMJ1a) and detected tentatively in wood- and microbial mat–containing samples from flowing and dry streams (FlowMG1b, FlowWJ1a, FlowMJ1c, FlowWJ1b, DryMJ1b) and bank sediments from the flowing stream (FlowBS1, FlowBS2). Methane responses were confirmed in microbial mat–containing samples from flowing and dry streams (FlowMG1a, FlowMJ1a, FlowMG2a, DryMJ1a) and detected tentatively in wood- and microbial mat–containing samples from the flowing stream (FlowWG1a, FlowWG1b, FlowMG2b) and bank sediments from the flowing stream (FlowBS1, FlowBS2). All confirmed hydrocarbon signals were accompanied by a methane signal, and the strength of methane responses tended to correlate with the hydrocarbon response. Only one wood-containing sample from the flowing stream (FlowWG1b) produced a methane peak where there was no measurable broad hydrocarbon feature. Water responses were confirmed for all samples. The most significant water response was observed for the microbial mat–containing sample from the flowing stream (FlowMG1a; 7.3% of the initial sample mass). Carbon dioxide responses were confirmed for all samples. Sulfur dioxide signals were absent in only three samples, namely, the microbial mat–rich samples from the flowing stream (FlowMG2a, FlowMG2b, and FlowMJ1b). Sulfur dioxide signals for the remaining samples were all significant.

**Table 3.** 1000°C Pyrolysis-FTIR Analysis

			*wt%*								
*Sample*	*Hydrocarbon absorbance* *± 0.02*		*Methane* *± 0.009*		*Water* *± 0.16*		*Carbon dioxide* *± 0.05*		*Sulfur dioxide* *± 0.04*		*Score*
FlowMG1a	1.29	C	1.202	C	7.30	C	7.57	C	0.35	C	A^*^AB
DryMJ1a	0.14	C	0.120	C	6.57	C	8.81	C	0.17	C	A^*^AB
FlowMJ1a	0.07	C	0.024	C	4.67	C	11.44	C	0.54	C	A^*^AB
FlowWG1a	0.05	C	0.009	T	4.11	C	8.04	C	0.26	C	A^*^AB
FlowMG2a	0.12	C	0.087	C	6.86	C	13.99	C	0.03	N	A^*^AC
FlowMG2b	0.04	C	0.016	T	3.77	C	4.59	C	-0.01	N	A^*^AC
FlowMG1b	0.02	T	-0.004	N	5.39	C	7.59	C	4.34	C	BAA
FlowWJ1a	0.02	T	0.006	N	4.16	C	4.29	C	5.74	C	BAA
FlowMJ1c	0.03	T	0.002	N	2.92	C	3.08	C	3.09	C	BAA
FlowBS2	0.03	T	0.017	T	3.95	C	2.90	C	1.51	C	BAA
DryMJ1b	0.03	T	-0.013	N	4.77	C	2.57	C	3.85	C	BAA
FlowWJ1b	0.03	T	-0.007	N	4.08	C	1.57	C	4.47	C	BBA
FlowWG1b	0.00	N	0.017	T	0.82	C	0.88	C	1.10	C	BBA
FlowBS1	0.02	T	0.017	T	2.30	C	1.11	C	0.14	C	BBB
FlowMG2c	0.01	N	-0.004	N	1.52	C	0.98	C	1.48	C	CBA
FlowBS3	0.00	N	0.003	N	2.29	C	0.75	C	0.31	C	CBB
FlowMG1c	0.01	N	-0.005	N	1.30	C	0.69	C	0.08	C	CBB
FlowQ1	0.00	N	0.008	N	1.09	C	0.54	C	0.13	C	CBB
FlowMJ1b	0.00	N	0.005	N	0.46	C	0.43	C	0.03	N	CBC

C = confirmed detection; T = tentative detection; N = null detection.

The samples in Table 3 were ranked by their scores following the 1000°C phase. Within each scoring group, ranking was determined by the signal strengths of the confirmed gas in the most important relevant tier (*i.e.,* Tier 1 > Tier 2 > Tier 3). In practice, this stage could allow a number of samples to be disregarded for further analysis. For the sake of this study, all samples were passed forward to phase two to reveal the robustness of the triage concept.

### 3.3. Triage phase two (the habitation assessment phase) using single-step pyrolysis-FTIR (700°C)

Results from the second triage phase (700°C single-step pyrolysis-FTIR) are presented in Table 4. Hydrocarbon responses were confirmed for the wood-, microbial mat–, and bank sediment–containing samples from flowing stream and microbial mat–containing samples from the dry stream (FlowWG1a, FlowMG1a, FlowMJ1a, FlowMJ1c, FlowMG2a, FlowMG2b, FlowBS1, FlowBS2, and DryMJ1a). For samples where hydrocarbons were tentatively detected at 1000°C and then confirmed at 700°C, those samples were promoted to A* for their Tier 1 organic response. These samples were derived from a microbial mat–containing sample and bank sediments from the flowing stream (FlowMJ1c, FlowBS1, FlowBS2). Hydrocarbons were detected tentatively at 700°C in wood-, mat-, and quartz sand–containing samples from the flowing stream and a mat-containing sample from the dry stream (FlowWJ1a, FlowWJ1b, FlowWG1b, FlowMG1b, FlowMG2c, FlowMJ1b, FlowQ1, DryMJ1b). The mat-containing sample from the flowing stream FlowMG1a was the only sample where a methane response could be confirmed. Methane responses were only tentatively observed in microbial mat–rich samples from the flowing and dry streams (FlowMG2a, FlowMG2b, DryMJ1a); the remaining samples had no measurable methane peak. Water and carbon dioxide were confirmed to be produced for all samples. Sulfur dioxide could not be detected from the pyrolysis of some wood- and microbial mat–containing samples from the flowing stream (FlowWG1b, FlowMG2a, FlowMG2b, FlowMJ1b) but was tentatively detected in the quartz sand from the flowing stream (FlowQ1) and confirmed for all other samples.

**Table 4.** 700°C Single-Step Analysis

			*wt%*									
*Sample*	*Hydrocarbon absorbance* *± 0.014*		*Methane* *± 0.03*		*Water* *± 0.03*		*Carbon dioxide* *± 0.07*		*Sulfur dioxide* *± 0.06*		*700°C*	*Score* ***700°C + 1000°C***
FlowMG1a	2.477	C	0.13	C	7.85	C	6.03	C	0.30	C	A^*^AB	A^*^AB
FlowMJ1a	0.311	C	0.03	N	4.57	C	7.01	C	0.64	C	A^*^AB	A^*^AB
DryMJ1a	0.263	C	0.05	T	6.09	C	5.32	C	0.19	C	A^*^AB	A^*^AB
FlowWG1a	0.172	C	0.02	N	4.64	C	6.89	C	0.24	C	A^*^AB	A^*^AB
FlowMG2a	0.194	C	0.06	T	6.95	C	8.23	C	0.04	N	A^*^AC	A^*^AC
FlowMG2b	0.082	C	0.04	T	4.63	C	4.32	C	−0.01	N	A^*^AC	A^*^AC
FlowBS2	0.079	C	0.01	N	3.60	C	1.22	C	1.89	C	A^*^BA	A^*^BA
FlowMJ1c	0.075	C	0.00	N	3.52	C	1.47	C	3.14	C	A^*^BA	A^*^BA
FlowBS1	0.075	C	0.01	N	2.34	C	0.49	C	0.16	C	A^*^BB	A^*^BB
FlowMG1b	0.022	T	−0.01	N	5.73	C	5.95	C	4.50	C	BAA	**BAA**
FlowWJ1a	0.026	T	−0.01	N	4.13	C	2.79	C	4.57	C	BAA	**BAA**
DryMJ1b	0.024	T	−0.01	N	5.47	C	2.28	C	3.23	C	BAA	**BAA**
FlowWJ1b	0.020	T	−0.02	N	4.59	C	1.38	C	2.48	C	BBA	**BBA**
FlowMG2c	0.026	T	0.00	N	1.56	C	0.66	C	1.23	C	*BBA*	**BBA**
FlowWG1b	0.019	T	0.00	N	0.73	C	0.35	C	0.03	N	*BBC*	**BBA**
FlowQ1	0.023	T	0.01	N	0.71	C	0.18	C	0.08	T	*BBB*	**BBB**
FlowMJ1b	0.016	T	0.01	N	0.48	C	0.34	C	0.05	N	*BBC*	**BBC**
FlowBS3	0.008	N	0.00	N	1.91	C	0.32	C	0.33	C	CBB	**CBB**
FlowMG1c	0.010	N	0.00	N	1.19	C	0.43	C	0.89	C	CBB	**CBB**

C = confirmed detection; T = tentative detection; N = null detection. Scores based on 700°C phase results alone are displayed in the left nonbold text column (cases for which the score differed from the 1000°C are italicized) and then combined with data from the previous 1000°C phase to give final scores (700°C + 1000°C) that are displayed in the right bold text column.

The scoring of samples at the 700°C phase was largely unchanged from the scoring in the 1000°C phase, except for a few notable exceptions.

(1) FlowMJ1a became a higher-priority sample than DryMJ1a owing to a stronger hydrocarbon response being revealed at 700°C.
(2) FlowBS2, FlowMJ1c, and FlowBS1 were all promoted from Tier 1 “B type” samples to “A* type” samples, as the presence of hydrocarbons could be confirmed at 700°C.
(3) FlowMG2c, FlowMJ1b, and FlowQ1 were both promoted from Tier 1 “C type” samples to “B type” samples, because hydrocarbons were tentatively detected at 700°C.
(4) Considering just the results of the 700°C step alone, FlowWG1b would be scored as a Tier 3 “C type” sample, because the sulfur dioxide signal was no longer present. However, as information of the previous triage step can be retained and scores are only improved as new detections are made, FlowWG1b ultimately retains a Tier 3 score of “A.” This was the only case where a sulfur dioxide signal, previously detected at 1000°C, was not detected at 700°C.

From these results, the highest-priority samples that were selected for improved characterization by multistep pyrolysis-FTIR were wood- and microbial mat–containing samples from flowing stream and a microbial mat–containing sample from the dry stream (FlowMG1a, FlowMJ1a, FlowMG2b, FlowMG2b, FlowWG1a, FlowWG1a, and DryMJ1a). The low-priority samples chosen to test the robustness of the triage concept were FlowMJ1b (the lowest-ranked sample) and FlowMG1c (a sample located from the same core as the highest-priority sample but representing a low-priority sample observed to produce all gases except hydrocarbons).

### 3.4. Triage phase three (the diagnostic phase) using multistep pyrolysis-FTIR (500°C, 750°C, and 1000°C)

The results from the third triage phase (multistep pyrolysis-FTIR) are presented in Table 5. Hydrocarbons were confirmed for all high-priority samples at the 500°C step but were absent from the 750°C and 1000°C steps, except for a tentative detection in a sample containing microbial mat over goethite from the flowing stream (FlowMG1a) at 750°C. For the low-priority comparison samples, hydrocarbon signals were absent in all analyses except for in a sample containing microbial mat over jarosite from the flowing stream (FlowMJ1b), where a hydrocarbon absorbance was tentatively observed. Methane was mostly undetected in all samples during multistep pyrolysis-FTIR, across all temperature steps, the exceptions being samples containing (i) microbial mat over goethite from the flowing stream (FlowMG1a) with methane observed tentatively at 500°C and confirmed at 750°C; (ii) microbial mat over jarosite from the dry stream (DryMJ1a) with methane observed tentatively at 500°C and 750°C; and (iii) microbial mat over goethite from the flowing stream (FlowMG2a) with methane observed tentatively at 750°C. Water was confirmed for all high-priority samples at 500°C and 750°C. For all these samples, quantities of water were on the order of 5 times greater at the lower of these two temperature steps, except for the microbial mat over jarosite sample from the dry stream (DryMJ1a), which had only about twice as much water at 500°C than 750°C. Water could only be confirmed for one high-priority sample at the 1000°C step, namely, the microbial mat over jarosite from the dry stream DryMJ1a. However, two samples containing microbial mat over goethite from the flowing stream (FlowMG1a and FlowMG2a) had tentative releases. The low-priority samples both produced confirmed water signals at 500°C and 750°C but no water responses at 1000°C. Carbon dioxide was confirmed in all cases, except for a sample of microbial mat over goethite from the flowing stream (FlowMG1c) at 1000°C. Sulfur dioxide could be detected in all samples at each temperature step. The majority of sulfur dioxide was observed at 750°C in each sample with the exception of samples of microbial mat over goethite and jarosite from the flowing stream (FlowMJ1a and FlowMG2b) where the main releases were at 500°C and 1000°C, respectively. In certain wood- and microbial mat–containing samples (FlowMG2a, FlowMG2b, FlowWG1a), no mineral source of sulfur was obvious, and the sulfur dioxide observed was likely to be biologically sourced. Following multistep pyrolysis-FTIR, the most shallow sample of microbial mat over goethite from the flowing stream (FlowMG1a) remained the highest-priority sample.

**Table 5.** Multistep Analysis, Performed on the Six Highest-Priority Samples Identified through the Preceding “Habitation Sensitivity” Triage Phase (Table 4)

			*% wt*							
*Sample*	*Hydrocarbon absorbance*		*Methane*		*Water*		*Carbon dioxide*		*Sulfur dioxide*	
*500°C*										
FlowMG1a	3.022		0.03		6.90		3.59		0.07	
DryMJ1a	0.363		0.03		4.49		3.26		0.13	
FlowMG2a	0.303		0.00		7.04		6.68		0.07	
FlowWG1a	0.075	± 0.013	0.01	± 0.02	4.28	± 0.09	3.94	± 0.05	0.29	± 0.03
FlowMJ1a	0.059		0.00		1.63		1.39		0.24	
FlowMG2b	0.035		0.00		3.83		2.41		0.00	
FlowMJ1b	0.024		−0.01		0.43		0.33		0.07	
FlowMG1c	0.006		0.00		1.01		0.30		0.13	
*750°C*										
FlowMG1a	0.05		0.11		1.24		3.86		0.34	
DryMJ1a	−0.01		0.04		2.13		4.38		0.23	
FlowMG2a	0.01		0.02		1.10		5.72		0.25	
FlowWG1a	0.00	± 0.03	0.01	± 0.02	0.90	± 0.11	4.12	± 0.03	0.49	± 0.03
FlowMJ1a	0.01		0.01		0.29		1.29		0.15	
FlowMG2b	0.00		0.01		0.60		2.51		0.14	
FlowMJ1b	0.01		0.01		0.71		0.49		0.06	
FlowMG1c	0.01		0.00		0.37		0.30		0.71	
*1000°C*										
FlowMG1a	0.005		0.01		0.25		3.42		0.29	
DryMJ1a	0.014		0.02		0.43		2.75		0.38	
FlowMG2a	0.014		0.02		0.19		4.89		0.40	
FlowWG1a	0.009	± 0.016	0.01	± 0.03	0.10	± 0.18	1.10	± 0.09	0.65	± 0.02
FlowMJ1a	0.003		0.00		0.08		0.75		0.15	
FlowMG2b	0.011		0.01		−0.06		0.57		0.21	
FlowMJ1b	−0.014		0.00		0.11		0.44		0.05	
FlowMG1c	−0.002		0.00		0.08		0.04		0.17	

Results are ordered by the total response of hydrocarbons for each sample across all three temperature steps. Two low-priority samples, FlowMJ1b and FlowMG1c, are included for comparison.

## 4. Discussion

### 4.1. Attenuated total reflectance-FTIR

Attenuated total reflectance results are used only as supporting data and would not be expected to be employed during sample triage on Mars Sample Return. The presence of water in all samples is perhaps expected considering their origin from a stream environment. The samples exhibiting hydroxyl peaks in ATR-FTIR correlate with samples that contain clay minerals which have hydroxyl units in their structure (Giese and Datta, 1973). Stronger hydroxyl signals tended to be observed in samples taken from near the edges of the flowing stream. Thus, the absence of hydroxyl peaks from a number of samples with biogenic matter above goethite or jarosite minerals (FlowMG1a, FlowMG1b, FlowMG2a, FlowMG2b, FlowMG2c, FlowMJ1c, FlowWJ1a, and DryMJ1b) derived generally from the middle of the stream suggesting that ATR results are reflecting variations in clay mineral contents at the various sampling locations. All samples exhibiting the broad 1400 cm^−1^ peak were also identified to have organic matter because alkanes, alcohols, and amides can have strong peaks in this region. There are a number of samples for which ATR-FTIR can identify features suggestive of sulfate bonds; one example is FlowMG2a (a sample of a microbial mat over goethite-rich minerals). FlowMG2a has a clear shoulder around 1090 cm^−1^, which is where a prominent sulfate peak occurs. It could be the case that sulfur compounds are present in noncrystalline forms within the biomass component of some samples.

### 4.2. Triage phase one (the habitability assessment phase) using single-step pyrolysis-FTIR (1000°C)

A sample from the flowing acid stream with wood fragments over goethite minerals (FlowWG1a) appears to show hydrocarbon signals at 1000°C, but hydrogen chloride peaks appear in the same region, and the assignment is uncertain. There is an apparent correlation between the strength of the hydrocarbon signal and the water signal. The weakest water response at 0.46% wt was from FlowMJ1b, the interior of the hard, sandy nodule, which has likely been isolated from aqueous processes for some time. FlowWG1b produced relatively small amounts of water and sulfur dioxide. It is probable that the most significant carbon dioxide signals are the by-products from organic compound combustion. For low-level responses, adsorbed species may contribute. Yet some samples produce no organic compound signals where the carbon dioxide signal is too significant to be attributed entirely to adsorbed species (*e.g.,* FlowMG2c, approx. 1% wt carbon dioxide); thus the carbon dioxide signal can act as nonspecific indicator for the presence of organic compounds in samples.

Particular attention should be paid to cases where organic compound signals are absent, the carbon dioxide signal is high, and there is a sulfur dioxide response, because of the ability of sulfates to produce oxygen during thermal decomposition, which can then combust organic matter (Lewis *et al.,* 2014). Three scenarios can be envisaged as follows: (i) sulfates are present in amounts greater than those needed to combust any organic matter, and carbon dioxide and sulfur dioxide are observed following pyrolysis, but no hydrocarbon signal exists; (ii) sulfates are present in amounts equal to those needed to combust any organic matter, and carbon dioxide is observed following pyrolysis, but no sulfur dioxide or hydrocarbon signals are present; and (iii) sulfates are present in amounts less than those needed to completely combust any organic matter, and carbon dioxide and organic responses are observed following pyrolysis, but no sulfur dioxide is present.

The most shallow and middle-depth samples from the least acidic core of the stream bed produced no sulfur dioxide, and samples from the edges of the stream produced relatively low levels of sulfur dioxide, indicating the influence of the sulfate-rich flowing water in sulfate mineral production. FlowMG2a had the strongest carbon dioxide signal of all samples, lacked a sulfur dioxide response, and had a hydrocarbon response. These data are consistent with scenario (iii) described above where carbon dioxide is enhanced and hydrocarbon signals diminished when organic matter is pyrolyzed in the presence of sulfates (Lewis *et al.,* 2014). For this sample, ATR-FTIR data hint at a possible sulfur feature.

The presence of water, carbon dioxide, and sulfur dioxide in pyrolysis-FTIR results can indicate habitable environments (Gordon and Sephton, 2016b). Single-step 1000°C pyrolysis-FTIR data were employed for habitability assessment. During single-step 1000°C pyrolysis-FTIR, FlowWG1b and FlowMJ1b were revealed as the least likely samples to have undergone significant alteration by water (water levels are 0.82% wt and 0.46% wt, respectively). Most other samples produced water >1% and thus can be suspected to contain hydrated mineral phases and are good candidates for habitable environments. It has been demonstrated that hydrated minerals generally produce water in amounts greater than 5% of mineral weight (Gordon and Sephton, 2016b). Carbon dioxide was observed for all samples, and the strongest carbon dioxide signal arose from FlowMG2a, which produces 14% carbon dioxide (pure carbonates produce carbon dioxide in amounts >20% of their weight). A number of samples produce significant sulfur dioxide signals (>1%) and thus can be assumed to contain sulfates. Methane production was confirmed for several samples: FlowMG1a (1.202%), DryMJ1a (0.120%), FlowMJ2a (0.024%), and FlowMG2a (0.087%). These samples were all obtained from the top of the respective cores where microbial mats were observed. Concurrent release of water and carbon dioxide can result from the combustion of hydrocarbons. Significant concurrent releases (both water and carbon dioxide >2% initial sample mass) were observed for FlowMG1a, FlowMJ1a, FlowWG1a, FlowMG2a, FlowMG2b, FlowMG1b, FlowWJ1a, FlowMJ1c, FlowBS2, DryMJ1a, and DryMJ1b. These signals were also generally stronger at the top of the cores where microbial mats are present. Strong concurrent releases are not observed for samples from the banks of the stream where microbial mats are absent. In summary, when using single-step pyrolysis at 1000°C, habitability is indicated by the release of water, carbon dioxide, or sulfur dioxide, while habitation is suggested by the concurrent release of carbon dioxide and water, and more strongly indicated by methane or more complex hydrocarbon signals.

### 4.3. Triage phase two (the habitation assessment phase) using single-step pyrolysis-FTIR (700°C)

When compared to the 1000°C single-step pyrolysis-FTIR phase, 700°C pyrolysis-FTIR produces stronger hydrocarbon signals across all samples tested. Consequently, three samples that were previously tentatively believed to have produced hydrocarbons were confirmed to produce hydrocarbons at the lower temperature (FlowBS2, FlowMJ1c, and FlowBS1). Four samples were promoted from producing no hydrocarbons to tentatively producing hydrocarbons (FlowMG2c, FlowWG1b, FlowQ1, and FlowMJ1b). FlowWG1b tentatively produced methane at 1000°C, so its score was unchanged. These results reinforce the choice of using 700°C as a more diagnostic temperature for detecting organic compounds than 1000°C. Across the entire sample set, methane signals were less intense following 700°C single-step pyrolysis-FTIR than during 1000°C single-step pyrolysis where almost complete conversion of all organic matter to a single analyte at the higher temperature facilitates detection.

Water quantities were observed to be similar between pyrolysis-FTIR analyses at 700°C and 1000°C, suggesting that the sources of water were probably the combustion of organic compounds, low energy bound mineral water (water of hydration and crystallization, associated with weathered material), and adsorbed water. Of the samples with strong hydroxyl responses from ATR-FTIR, only FlowBS1 and FlowWJ1b did not produce more water at 1000°C than at 700°C (FlowBS2, FlowBS3, and DryMJ1a all produced more water at 1000°C).

Carbon dioxide amounts produced from the samples were lower at 700°C than at 1000°C. The reason for lower levels of carbon dioxide at 700°C is because at 1000°C the extent of hydrocarbon combustion is greater. High-temperature carbon dioxide can result from decomposition of calcium carbonate, but any mineral source of carbon dioxide was eliminated by the ATR-FTIR data (Tables 1 and 2).

Sulfur dioxide quantities are generally comparable between the single-step 700°C and 1000°C pyrolysis-FTIR modes. For FlowWG1b, the signal seen at 1000°C disappears when subjected to pyrolysis at 700°C, and the sulfur dioxide signal for FlowQ1 (confirmed at 1000°C) was only tentatively observed. Samples with sulfur dioxide responses that were only observed when pyrolyzed at a higher temperature most probably contain more thermally stable sulfate minerals; for example, calcium, sodium, and magnesium sulfates all decompose above 700°C (Lewis *et al.,* 2014).

### 4.4. Triage phase three (the diagnostic phase) using multistep pyrolysis-FTIR (500°C, 750°C, and 1000°C)

Using data from the two previous phases of assessment (single step pyrolysis at 1000°C and 700°C), we were able to select the highest-priority samples (FlowMG1a, FlowMJ1a, DryMJ1a, FlowWG1a, FlowMG2a, and FlowMG2b) for improved characterization by multistep pyrolysis-FTIR. Two low-priority samples were also chosen for comparison (FlowMJ1b and FlowMG1c).

The three most organic-rich samples processed by multistep pyrolysis-FTIR (FlowMG1a, FlowMG2a, and DryMJ1a) produced their major hydrocarbon responses at 500°C and relatively constant quantities of carbon dioxide across all temperature steps. The three other high-priority samples displayed a reduction in carbon dioxide response at 1000°C to approximately half (FlowMJ1a) or a quarter (FlowWG1a and FlowMG2b) of that at 750°C. FlowMG1c had displayed the weakest response for organic compounds in previous assessments, and what organic matter that was present was detected at 500°C. The temperature resolution of multistep pyrolysis-FTIR appears highly diagnostic for the presence of organic matter.

For the samples with the poorest organic response (FlowMG1c), carbon dioxide was released at 500°C and 750°C but not at 1000°C. Sulfur dioxide was present at all temperatures, but the maximum release occurred at 750°C (0.71% wt). The temperature-step release profile was reflective of the temperature-dependent reactions of organic matter and sulfur dioxide. At 500°C, organic matter breaks down, but sulfate decomposition is not yet at peak activity. At 750°C, sulfate decomposition is maximal and is oxidizing organic matter to carbon dioxide. At 1000°C, sulfate decomposition is still occurring, though all organic matter has been oxidized, and no further carbon dioxide can be produced. The gas release profiles in multistep pyrolysis-FTIR of the organic-poor sample demonstrate its capability of isolating the release of gases from different sources with different decomposition temperatures thereby providing guidance for strategies that avoid secondary reactions that may otherwise obscure scientific information.

During multistep pyrolysis-FTIR, the majority of minerals released the bulk of their total water products at the 500°C temperature step, making weathered rock and clays a most likely source. FlowMG1a, DryMJ1a, FlowMG2a, and FlowWG1a still produced significant quantities of carbon dioxide at 1000°C (>1% wt). High-temperature carbon dioxide release is a signature of certain carbonates (*e.g.,* calcium carbonate), but in the acidic stream environment carbonates are unlikely to have formed, although it should be noted that ankerite, a calcium-bearing carbonate, has been observed to be precipitated by microbes in acidic environments (Fernández-Remolar *et al.,* 2012) and produces carbon dioxide at temperatures >800°C (Meurant 1967).

### 4.5. Assessment of the triage process

**FIG. 4.**
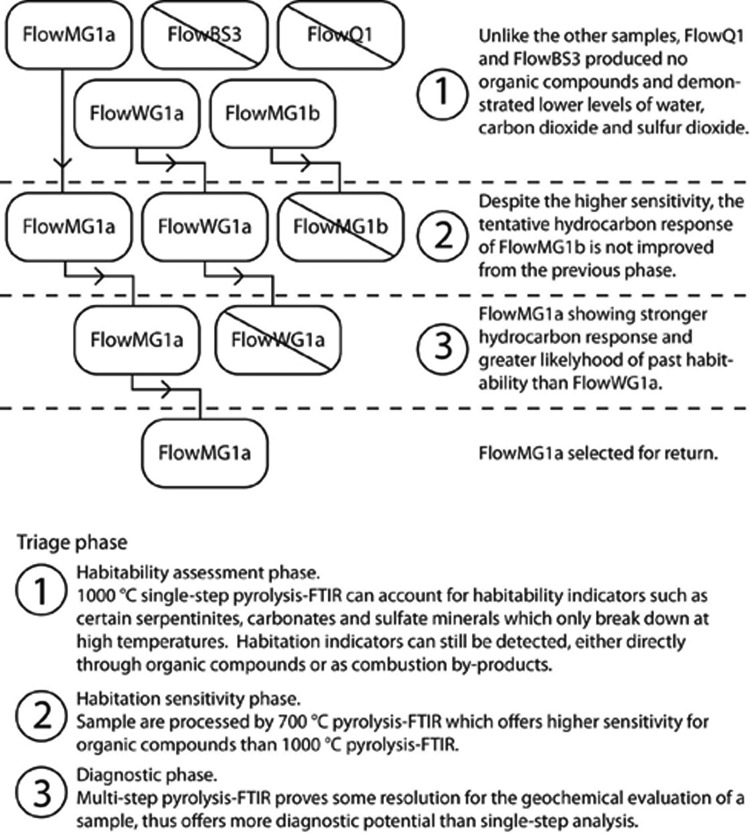
Example triage operation.

Figure 4 illustrates an example application of the triage operation on a subset of the samples used in this study, where resource limitations require that candidate samples are eliminated at each phase. In analogy to a Mars sample return mission, the priority would be to detect evidence of past or present life (McLennan *et al.,* 2012). To this end, the correctness of the sample ranking process was evident during the very first triage step using single-step pyrolysis-FTIR at 1000°C where the highest-priority samples were correctly ranked (Table 3). The pyrolysis-FTIR triage process clearly identified the biomass-rich FlowMG1a to be the highest-priority sample and assigned the relatively barren bank sediment FlowBS3 and deep core FlowMG1c to be the lowest-priority samples. It is notable that the scoring system prioritized the four microbial mat samples, and Flow MG1a, FlowMJ1a, FlowMG2a, and DryMJ1a routinely occupy four of the five highest positions. The next group of samples favored by the scoring system were those with mineral components relatively high in goethite, namely, FlowMG1b, FlowMG2b, and FlowWG1a (Table 3). The identification of the goethite-rich samples as high priorities is in accord with recently published data that reveal that goethite minerals derived from the humidity-assisted decomposition of jarosite display a high preservation potential for organic records (Lewis *et al.,* 2018). The pyrolysis-FTIR triage process also provided refinements to prioritizations during the second stage of triage using single-step pyrolysis-FTIR at 700°C. For example, FlowMJ1b was the lowest-ranked sample at 1000°C (Table 3), which was partly a result of having no detectable hydrocarbon signal, yet was then revealed to have a tentative hydrocarbon signal during the 700°C phase (Table 4) probably reflecting the attenuating effect of sulfate decomposition products on organic responses at higher temperatures. In a Mars context, the suspicion of organic signal attenuation would justify further characterization by multistep pyrolysis, during which a diagnostic hydrocarbon signal would have been observed in the 500°C step (Table 5). False negatives are outcomes that should be avoided in any Mars sample triage process, and it is notable that our triage process would recognize the organic matter–bearing samples, that is, our method has high sensitivity, because the potential presence of organic compounds can be recognized through the simultaneous releases of water and carbon dioxide.

The rapid analysis times involved in pyrolysis-FTIR make this triage method appropriate for high numbers of samples. The phased approach allows discrimination of low-value samples early in the procedure, which in turn improves efficiency of mission resources.

### 4.6. Additional considerations

Pyrolysis-FTIR requires that samples are crushed and delivered in a powdered form, a protocol common to previous lander and rover missions to Mars with life-detection goals (*e.g.,* Viking and Mars Science Laboratory). It should be noted that the analog samples are not representative of the complete history of Mars but are relevant to the Late Noachian and Early Hesperian where valley networks and sulfate deposits indicate acidic surface waters.

## 5. Conclusions

The success of Mars Sample Return will depend on the quality of collected materials, so samples must be assessed and prioritized for return to Earth. The quantitative chemical information produced by pyrolysis-FTIR is effective for ranking candidate samples based on criteria relevant to the search for life on Mars, namely, the habitability of the sample environment, the presence of organic compounds, and the preservation potential of the sample for biosignatures. A scoring system is developed based on the detection of (i) organic signals, (ii) carbon dioxide, and water and (iii) sulfur dioxide. The presence of each component is given a score of A, B, or C depending on whether the substance has been detected, tentatively detected, or not detected. Complex organic matter in Tier 1 is indicated by A*. The pyrolysis-FTIR triage operation benefited from a phased approach, where single-step pyrolysis-FTIR at 1000°C and single-step pyrolysis-FTIR at 700°C provide sensitivity, with the former producing bulk signals for habitability indicators and organic content across the broadest range of potential sources and the latter being the most sensitive to detecting organic compounds, while multistep pyrolysis-FTIR at 500°C, 750°C, and 1000°C and all three phases in conjunction offer specificity through added resolution.

## Abbreviations Used

ATR: attenuated total reflectance
FTIR: Fourier transform infrared
